# Supplementary figures and images for: Antimicrobial stewardship hindered by inadequate biosecurity and biosafety practices, and inappropriate antibiotics usage in poultry farms of Nepal–A pilot study
Source: PLoS One. 2024 Mar 1;19(3):e0296911. doi: 10.1371/journal.pone.0296911 (PMC10906820; doi:10.1371/journal.pone.0296911)

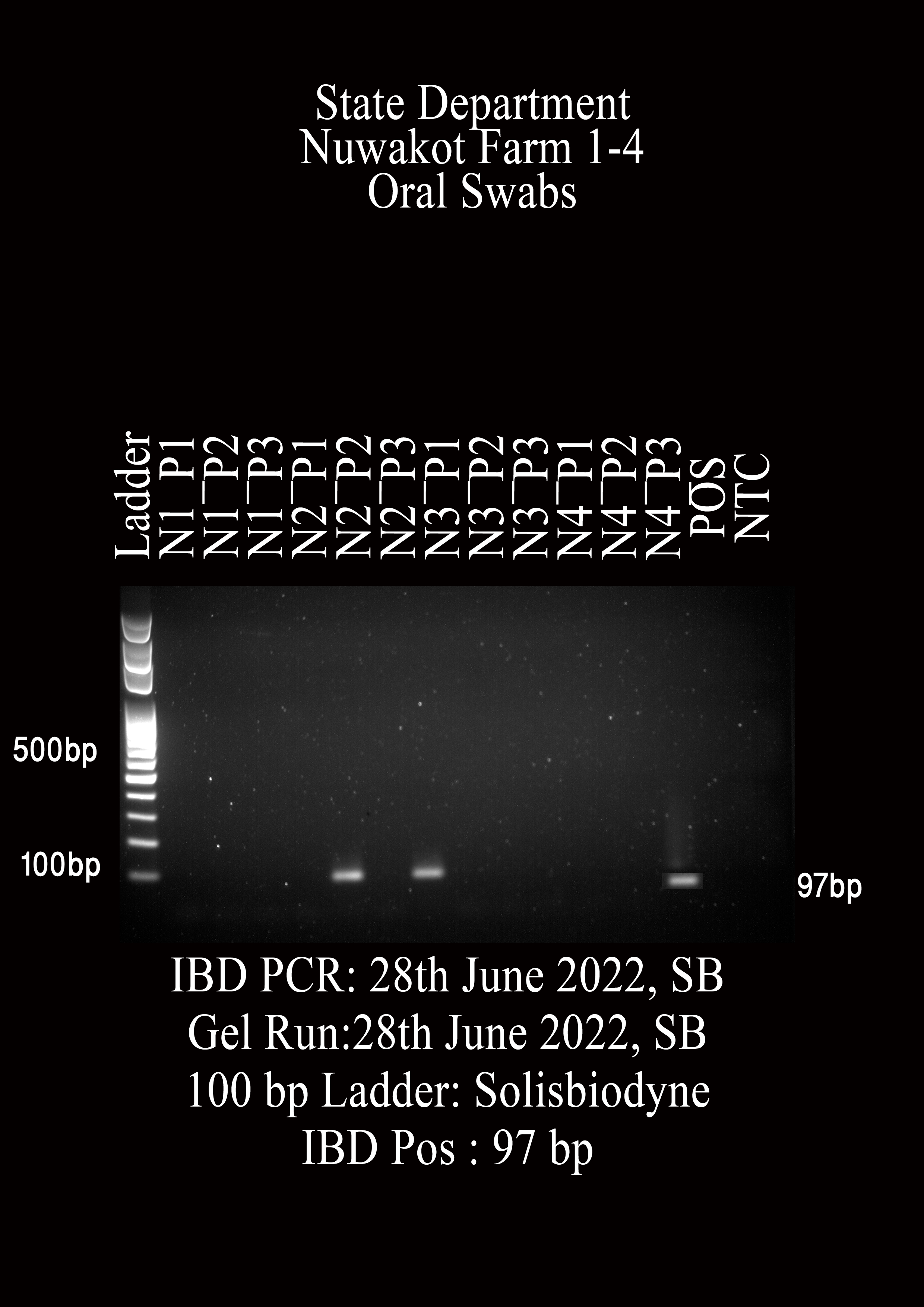

Supplement: S1 Fig — The four farms were numbered from N1 to N4. Each sample represents pooled oral and cloacal samples. The gel was run with ladder in the first well and positive and negative controls in the last two well respectively. (IBD): Infectious Bursal Disease (IBD) detected in poultry farms of Nuwakot District. The four farms were numbered from N1 to N4. Each sample represents pooled oral and cloacal samples. The gel was run with ladder in the first well and positive and negative controls in the last two well respectively. (ZIP) [file pone.0296911.s001.zip › Figure S1 - (IBD).tif]

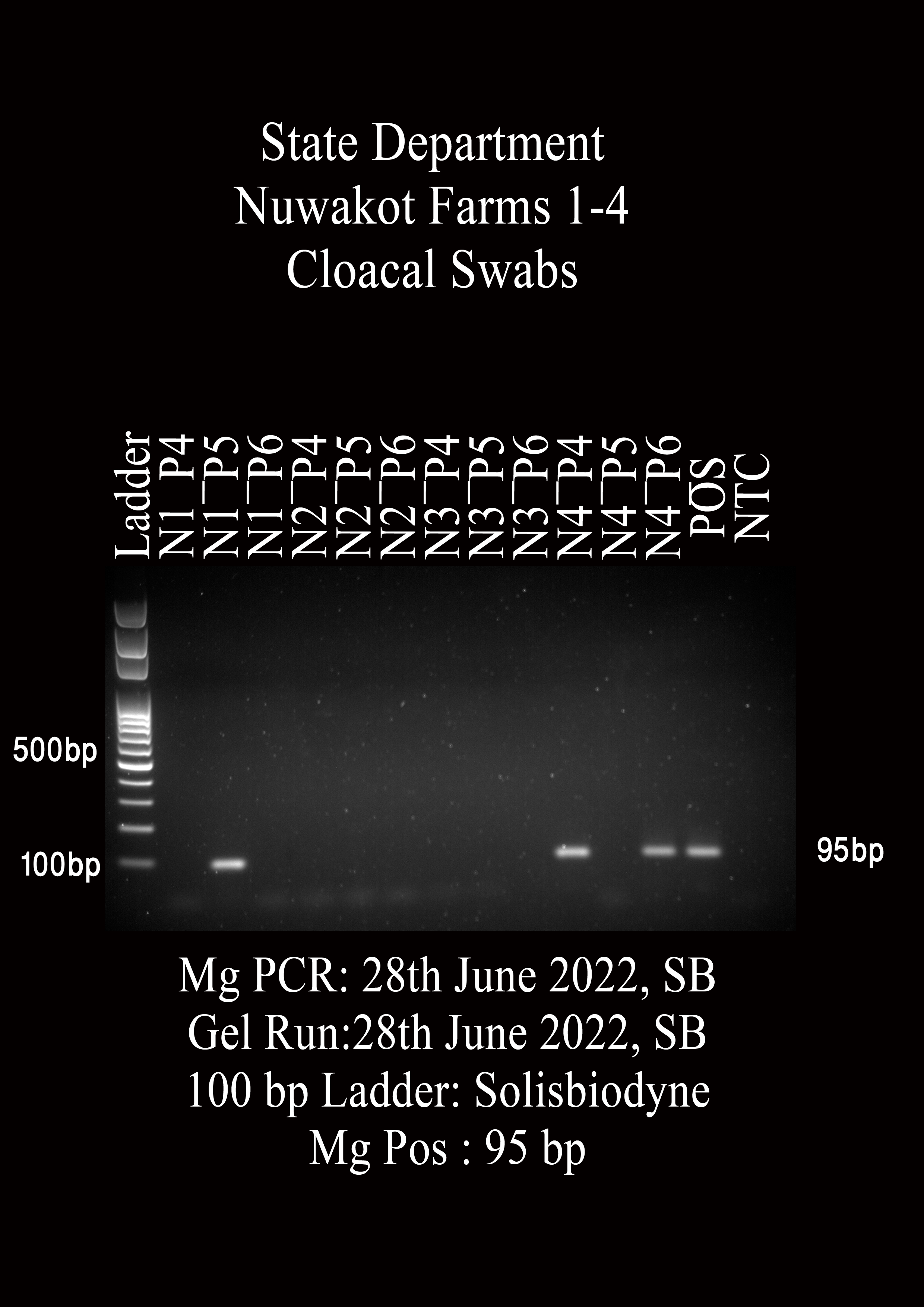

Supplement: S1 Fig — The four farms were numbered from N1 to N4. Each sample represents pooled oral and cloacal samples. The gel was run with ladder in the first well and positive and negative controls in the last two well respectively. (IBD): Infectious Bursal Disease (IBD) detected in poultry farms of Nuwakot District. The four farms were numbered from N1 to N4. Each sample represents pooled oral and cloacal samples. The gel was run with ladder in the first well and positive and negative controls in the last two well respectively. (ZIP) [file pone.0296911.s001.zip › Figure S1 - (Mg).tif]

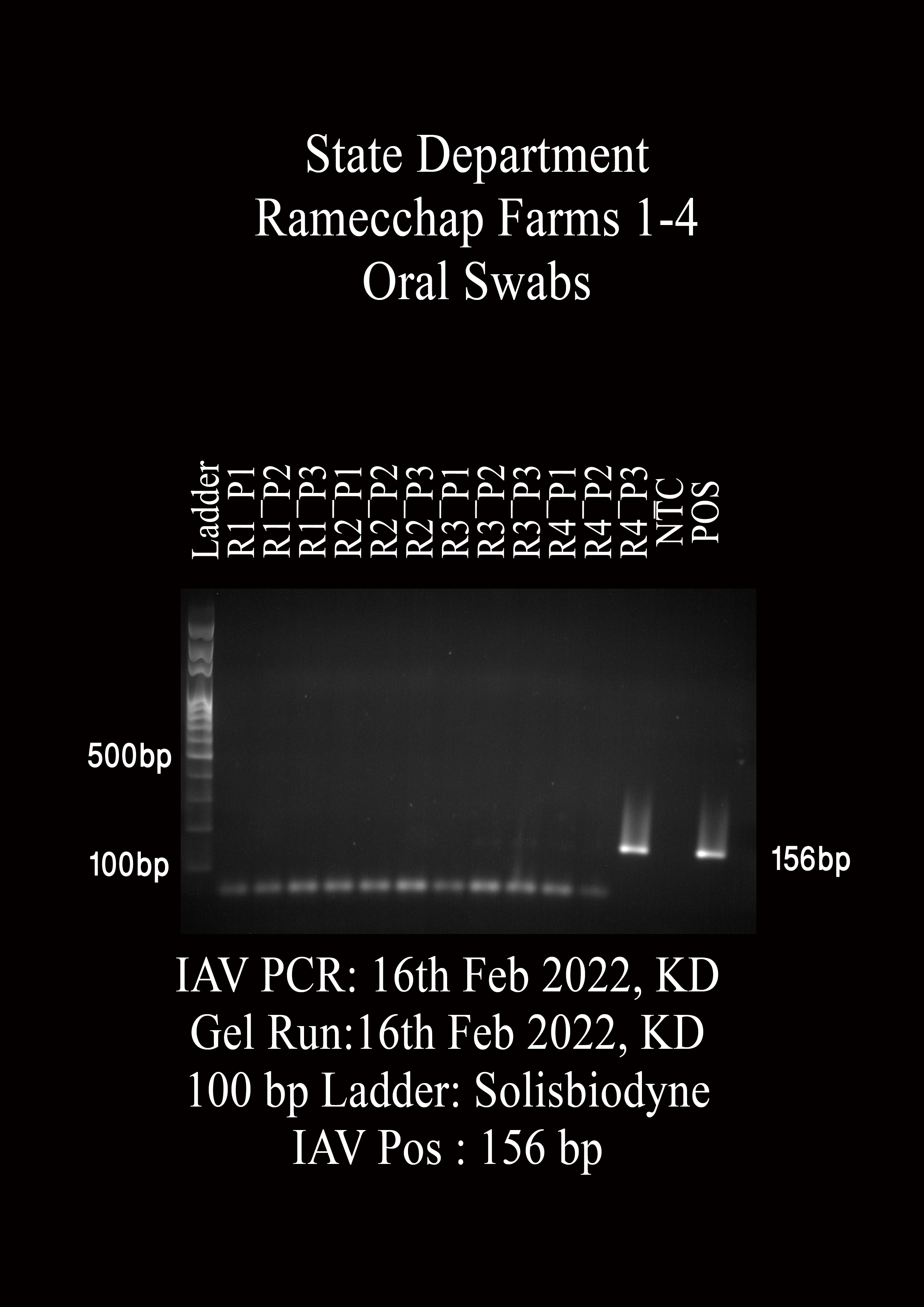

Supplement: S2 Fig — The four farms were numbered from R1 to R4. Each sample represents pooled oral and cloacal samples. The gel was run with ladder in the first well and positive and negative controls in the last two well respectively. (TIF) [file pone.0296911.s002.tif]

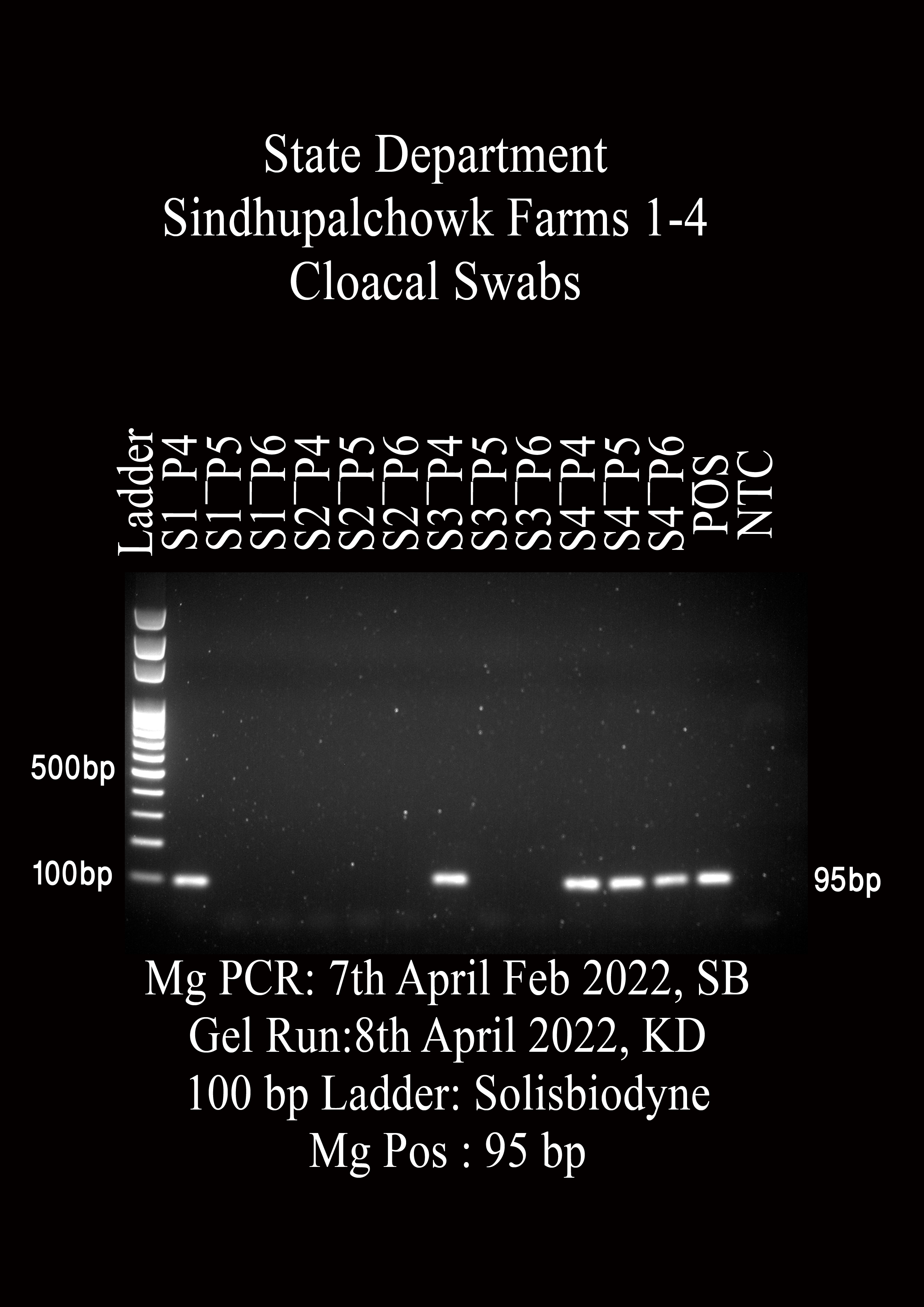

Supplement: S3 Fig — The four farms were numbered from S1 to S4. Each sample represents pooled oral and cloacal samples. The gel was run with ladder in the first well and positive and negative controls in the last two well respectively. (TIF) [file pone.0296911.s003.tif]

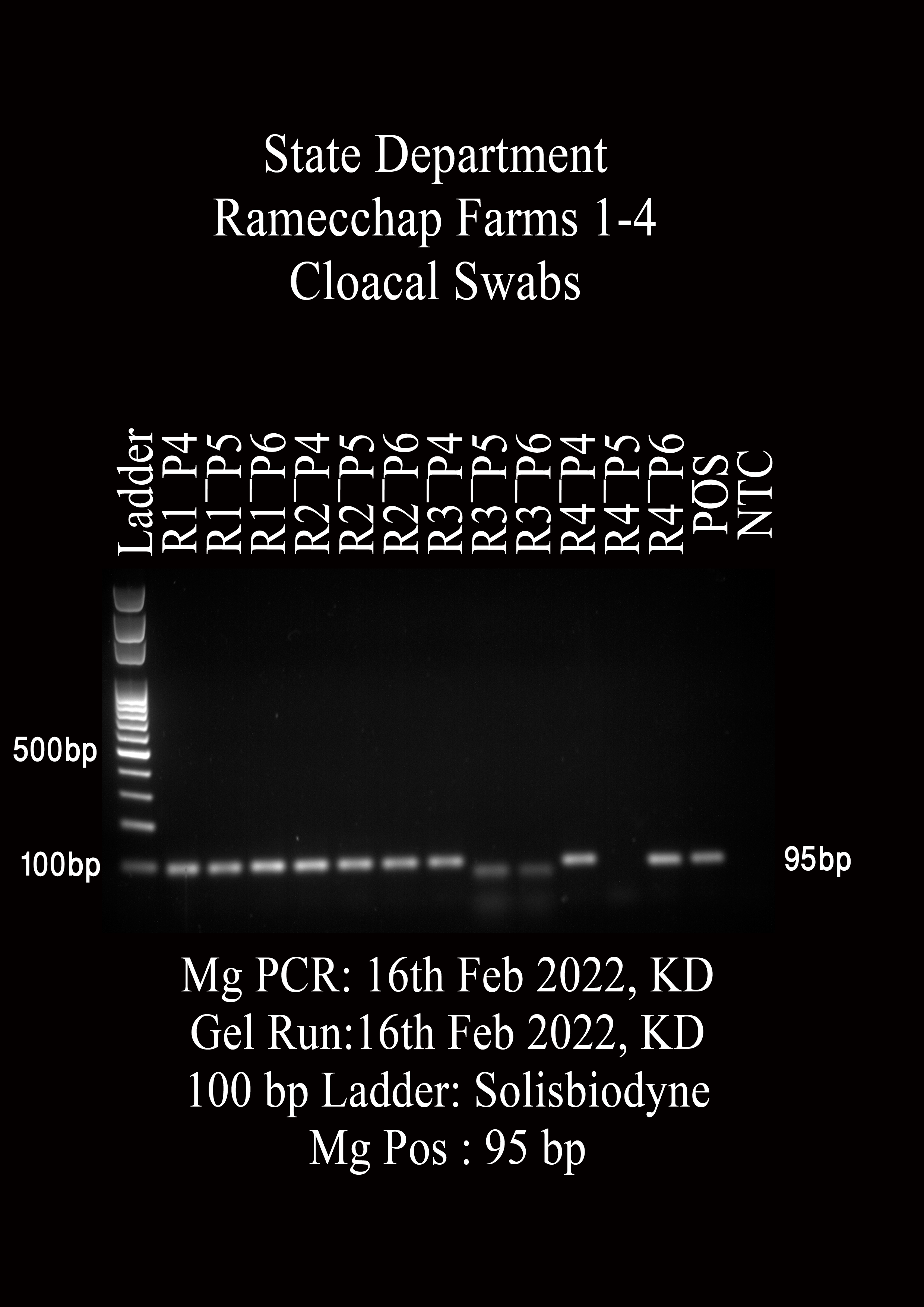

Supplement: S4 Fig — The four farms were numbered from R1 to R4. Each sample represents pooled oral and cloacal samples. The gel was run with ladder in the first well and positive and negative controls in the last two well respectively. (Ms): Mycoplasma synoviae (Ms) detected in poultry farms of Ramechhap District. The four farms were numbered from R1 to R4. Each sample represents pooled oral and cloacal samples. The gel was run with ladder in the first well and positive and negative controls in the last two well respectively. (ZIP) [file pone.0296911.s004.zip › Figure S4 - (Mg).tif]

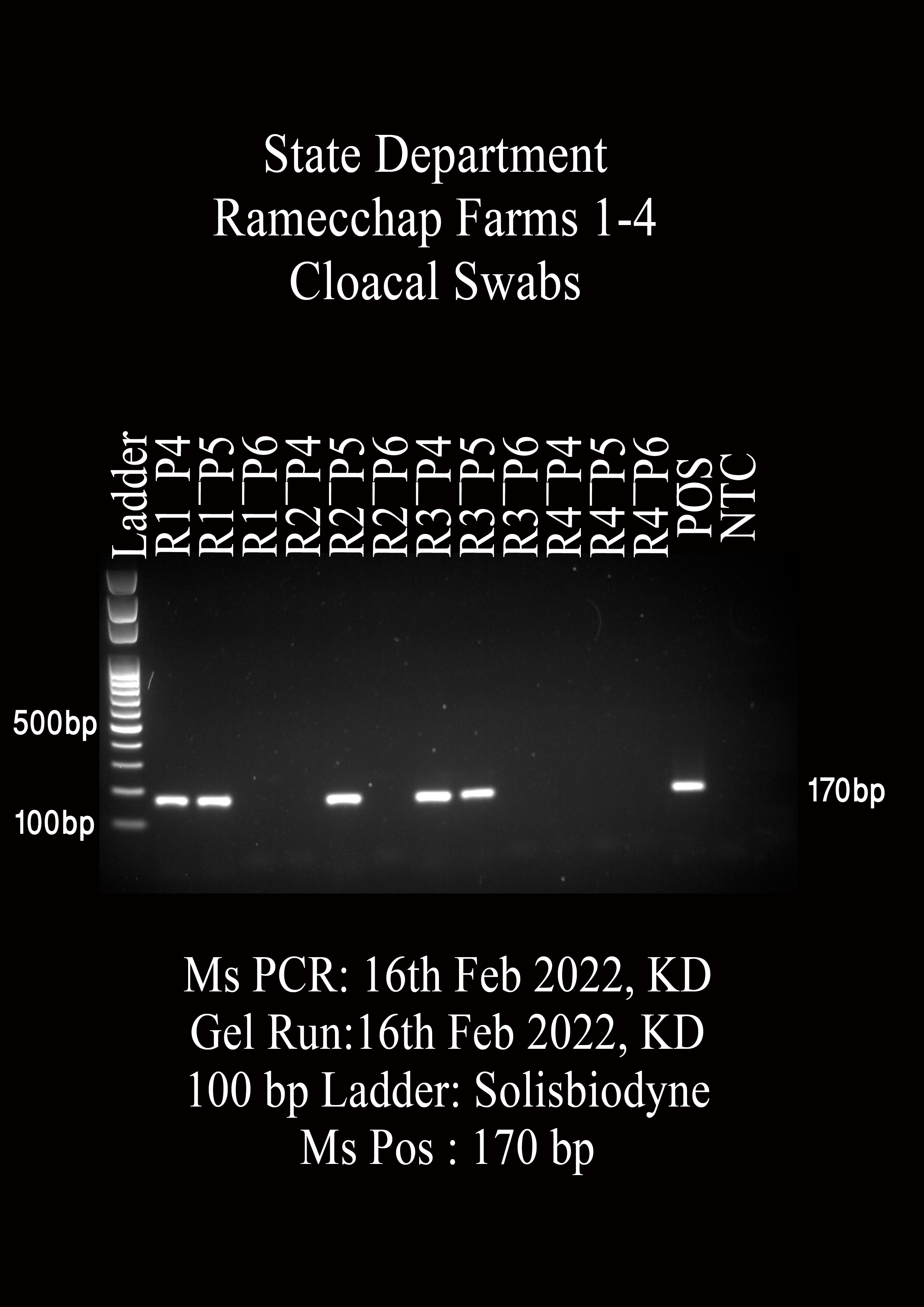

Supplement: S4 Fig — The four farms were numbered from R1 to R4. Each sample represents pooled oral and cloacal samples. The gel was run with ladder in the first well and positive and negative controls in the last two well respectively. (Ms): Mycoplasma synoviae (Ms) detected in poultry farms of Ramechhap District. The four farms were numbered from R1 to R4. Each sample represents pooled oral and cloacal samples. The gel was run with ladder in the first well and positive and negative controls in the last two well respectively. (ZIP) [file pone.0296911.s004.zip › Figure S4 - (Ms).tif]

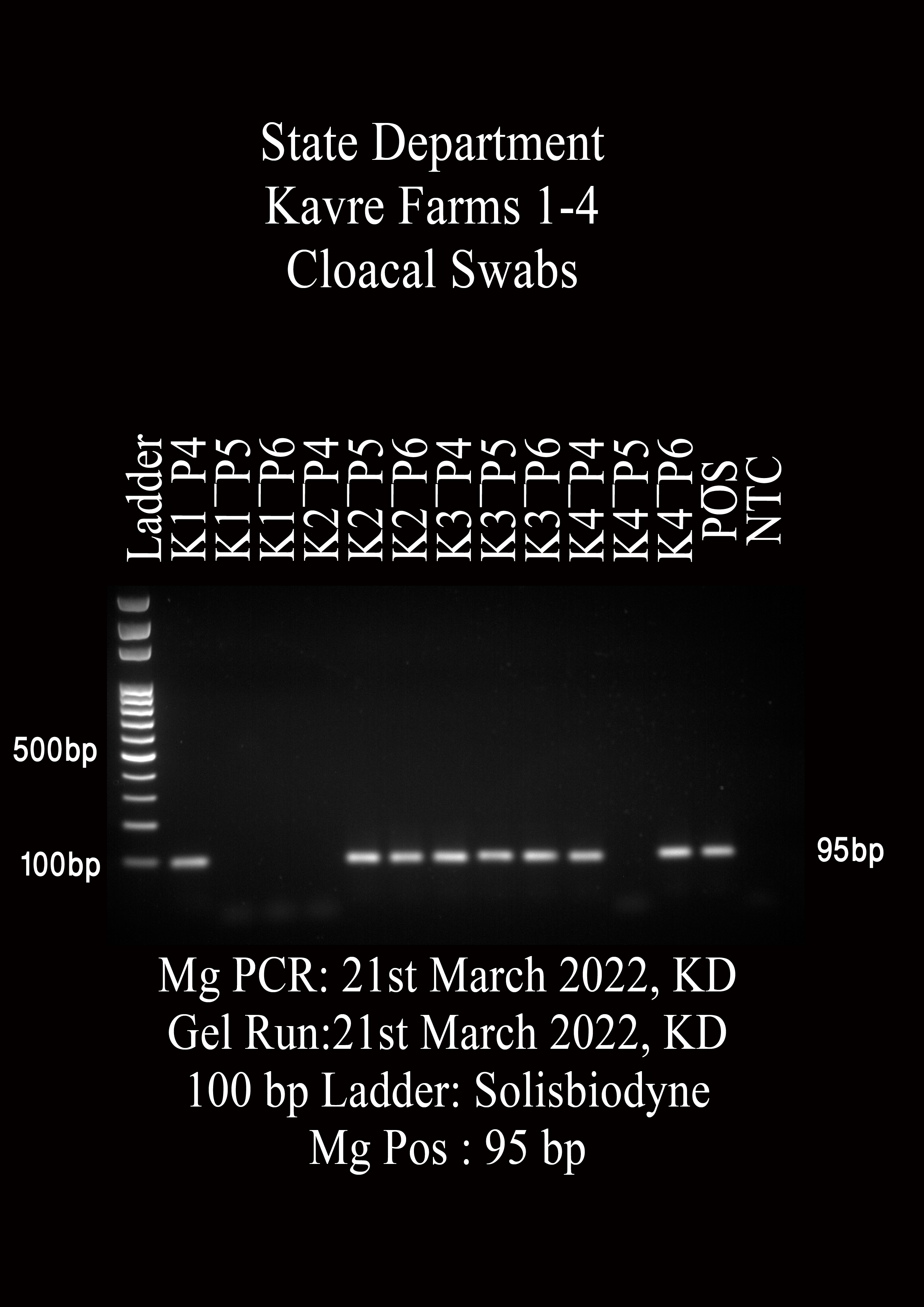

Supplement: S5 Fig — The four farms were numbered from K1 to K4. Each sample represents pooled oral and cloacal samples. The gel was run with ladder in the first well and positive and negative controls in the last two well respectively. (TIF) [file pone.0296911.s005.tif]
