# Supplementary material for: Antimicrobial stewardship hindered by inadequate biosecurity and biosafety practices, and inappropriate antibiotics usage in poultry farms of Nepal–A pilot study
Source: PLoS One. 2024 Mar 1;19(3):e0296911. doi: 10.1371/journal.pone.0296911 (PMC10906820; doi:10.1371/journal.pone.0296911)
Supplement: S1 Table — (DOCX) [file pone.0296911.s006.docx]

**Supplementary Table 1: Biosafety and biosecurity checklist used to assess the farms**

**BIOSECURITY CHEKCLIST FOR POULTRY FARM**

**Location: GPS: Date:**

**Recorded By:**

| **Activities** | **Completed** | **Remarks** |
| --- | --- | --- |
| ***General Practices*** |  |  |
| 1. Perimeter fence around the production area – defined biosecurity zone |  |  |
| 2. Any other animal or bird kept at the facility must be screened for diseases to avoid transmission |  |  |
| 3. Defined area for free-range poultry to graze |  |  |
| 4. Main entrance to the production must be capable of being closed off to other people/vehicle |  |  |
| 5. Defined area (away from shed) for parking, wearing PPE |  |  |
| 6. Entry into the shed must have a footbath or similar disinfectant available, provision for scraping boots/footwear |  |  |
| 7. Hand washing facility present near the shed |  |  |
| 8. A dedicated area for new birds and one for collection of dead birds |  |  |
| 9. Shed covered with mesh or tarp to prevent entry of wild birds |  |  |
| 10. Proper drainage system in the production area – avoid water stagnation and breeding area for diseases |  |  |
| 11. Baits for rodents or other pests if they frequent the farm |  |  |
| 12. Closed system for treated and sanitized water poultry |  |  |
| 13. No other birds kept in the production are apart from production birds |  |  |
| 14. If more than one type of production bird is present, they must be housed and managed separately, shared equipment disinfected between uses |  |  |
| 15. Backyard chickens kept away from production chickens |  |  |
| 16. Closed feeding systems, protected from access & contamination by wild birds and rodents |  |  |
| 17. Any feed spilled outside the shed cleaned immediately |  |  |
|  |  |  |
| ***Personnel Standards and Procedures*** |  |  |
| 1. Poultry production area staff must NOT have any contact with other poultry, cage birds, ostriches, and pigeons while actively engaged in the production area |  |  |
| 2. Staff must wear appropriate and clean PPE or on-farm clothing and footwear |  |  |
| 3. Staff must NOT move around the farm using the same PPE, one set must be dedicated for the shed only |  |  |
| 4. Apart from staff, any other personnel must avoid multiple trips to the production area on the same day. If they must, appropriate PPE must be worn |  |  |
| 5. Hands washed/sanitized before entering the shed |  |  |
| 6. Repair/maintenance personnel or any other outside personnel must not enter populated area of the shed unless it’s an emergency |  |  |
| 7. Routine maintenance and disinfection conducted between batches (for broiler chickens) |  |  |
| 8. Tools taken into production area/shed must be thoroughly cleaned and disinfected |  |  |
| 9. Records kept (logs) of all non-production staff personnel that visit the farm |  |  |
| 10. Any other colleague, friends, or family that might have come in contact with other poultry outside of the farm must not enter the shed |  |  |
| 11. Non-essential vehicles must be parked at least 30m from the production area |  |  |
| 12. Feed transfer crews and flock pick-up crews must NOT visit on the same day |  |  |
| 13. Pick up vehicles must be thoroughly cleaned |  |  |
| 14. Day-old-chick vehicles must be disinfected before offloading the chicks and before leaving the property |  |  |
| 15. Delivery personnel must sanitize their hands before offloading any material |  |  |
|  |  |  |
| **Activities** | **Completed** | **Remarks** |
| ***Water Supply*** |  |  |
| 1. Water disinfected before feeding the chickens |  |  |
| 2. When chlorinating, the water must have minimum of 2 hours of contact time with chlorine before use |  |  |
| 3, Water supply must be checked for cleanliness and disinfected daily |  |  |
| 4. Drinking water standards - colony count ≤ 1000, E. Coli – Nil, Coliforms ≤ 100 |  |  |
|  |  |  |
| ***Rodent Control*** |  |  |
| 1. Baits placed at regular intervals, more baits placed in regions of high rodent activities |  |  |
| 2. Baits checked weekly and replaced with fresh baits as needed |  |  |
| 3. Baits designed to prevent other animals apart from rodents/pests from accessing them |  |  |
| 4. Records kept of frequency of rodents |  |  |
|  |  |  |
| ***Cleaning and Maintenance*** |  |  |
| 1. Feed spills cleaned as soon as practicable as it attracts birds and rodents |  |  |
| 2. Grass on and around the production area are cut – long grass attracts rodents and favors survival of bacteria and viruses |  |  |
| 3. Footbaths inspected daily for excessive organic matter and replaced as required |  |  |
| 4. Free-range area adequately drained to avoid water stagnation and slope created to avoid influx of runoff water from other parts |  |  |
| 5. Manure and litter from poultry in adjacent land carefully considered to avoid spread of diseases |  |  |
| a. Dry cleaning – brushing, scraping, broom cleaning |  |  |
| b. Wet cleaning - soaking, washing, and rinsing using detergents and using washer with warm water for ceiling, walls, floors, platforms, equipment – to loosen debris and improve penetration of cleaning agents |  |  |
| c. Washing – washing every surface in the building using neutral detergent – done after wet cleaning |  |  |
| d. Disinfection – equipment disinfected within 24 hours of cleaning and drying |  |  |
| e. Disinfection – dependent on surfaces and pathogens targeted; usually work better at temperature above 20^O^C |  |  |
| f. PPE worn while cleaning, washing, and disinfecting |  |  |
|  |  |  |
| ***Record Keeping*** |  |  |
| 1. Daily feed and water consumption |  |  |
| 2. Daily bird mortality or sickness |  |  |
| 3. Any biosecurity breach |  |  |
| 4. Vaccinations |  |  |
| 5. Additional treatments (medications – date, duration, and outcomes) |  |  |
| 6. Visitors and vehicles logs |  |  |
|  |  |  |
| ***Others*** |  |  |
|  |  |  |
|  |  |  |
