# Supplementary material for: Antimicrobial stewardship hindered by inadequate biosecurity and biosafety practices, and inappropriate antibiotics usage in poultry farms of Nepal–A pilot study
Source: PLoS One. 2024 Mar 1;19(3):e0296911. doi: 10.1371/journal.pone.0296911 (PMC10906820; doi:10.1371/journal.pone.0296911)
Supplement: S2 Table — (DOCX) [file pone.0296911.s007.docx]

**Supplementary Table 2: Antibiotic stewardship survey used to assess farm owners’ knowledge on antibiotics and their usage.**

**ANTIBIOTIC STEWARDSHIP SURVEY FOR FARM OWNERS**

| **Questions** | **Remarks** |
| --- | --- |
| Knowledge on antimicrobials? (Y/N) |  |
| Prophylactic usage of antimicrobials? (Y/N) |  |
| Therapeutic usage of antimicrobials? (Y/N) |  |
| Conduction of post-mortem of dead animals? (Y/N) |  |
| Route for antimicrobials? Feed (F), Water (W), Injection (I)? |  |
| Knowledge on withdrawal period? (Y/N) |  |
| Purchase with prescription? (Y/N) |  |
| Withdrawal period prior to culling? (Y/N) |  |
| Knowledge on Antimicrobial Resistance? (Y/N) |  |
| Participation on Antimicrobial awareness programs? (Y/N) |  |
| Source of antibiotic knowledge? Local animal health centre (L), Veterinary doctor(D), Veterinary technician (T), Vet suppliers and shop(S), Own(O) |  |
| Last time antibiotic was used? This week (W), month(M), Year(Y), Never(N) |  |
| Perception of AMR as a threat? Y/N |  |
| More trustworthy stakeholder? Veterinary doctor(D), Vet technician (T), Local Animal Health Centre (L), Self-treatment(O), Shop and Sales (S)? |  |
| Most used antibiotics according to scale | Subjective question |
