# Supplementary material for: Antimicrobial stewardship hindered by inadequate biosecurity and biosafety practices, and inappropriate antibiotics usage in poultry farms of Nepal–A pilot study
Source: PLoS One. 2024 Mar 1;19(3):e0296911. doi: 10.1371/journal.pone.0296911 (PMC10906820; doi:10.1371/journal.pone.0296911)
Supplement: Attachment — Submitted filename: PONE-D-23-10881_R1_review report.pdf [file pone.0296911.s010.pdf]

The manuscript entitled "Antimicrobial stewardship hindered by inadequate biosecurity and biosafety practices, and inappropriate antibiotics usage in poultry farms of Nepal – A pilot study" describes the biosecurity gaps and antibiotic uses in poultry farms. The Authors identified poultry bacteria and viruses from those farms as well. Actually, they tried to show a relationship between poor biosecurity and antimicrobial use in poultry farms around Kathmandu City, Nepal.

The manuscript has importance in the field of biosecurity and AMR along with antimicrobial stewardship research in poultry farms. However, the manuscript requires some significant corrections before publication.

The title of the manuscript requires a change. The Authors used biosafety in the title and throughout the manuscript. According to WHO, **Biosafety is the safe working practices associated with handling of biological materials.** This is related to laboratories or any area where biological materials particularly, pathogens are handled. Therefore, to me, biosafety is not associated with poultry farms. I suggest erasing the word biosafety from the title as well as the whole manuscript.

General comment: Minor English collections are required, particularly, grammar and spelling checks. The table and figure should be self-explanatory. Species should be italicized. Please check the references are appropriately selected. Find the other corrections and clarifications below.

Line No. 22-24: Please check the sentence, particularly this part, "can complicate the spread of poultry diseases…". Maybe it will be, "can enhance the spread of poultry diseases…."

Line no. 70-71: Please mention, for which animal feed unprocessed poultry waste is being used and contributes to the spread of AI. Please check the reference (13) as well for this statement in line no. 70-71.

Line no. 73: Please check the format of writing *Escherichia coli* and make the first letter of salmonella as capital.

Line no. 86-87: The Authors have justified the cause of the sampling area around the city of Kathmandu. They used a map showing the sample collection area (four districts, including the farms in the districts) for a better understanding of how the city is surrounded by sample collection areas. I suggest adding the Figure 1 in the methods sections.

Line no. 106-107: Marek's disease virus detection from feather follicle epithelial cells is best. There are some other important samples like chicken dust, bedding material, etc. Why the Authors screened Marek's disease virus from cloacal and oropharyngeal swabs?

Please mention the age of every farm selected for sample collections.

Line no. 151-152: What is the basis of the categorization as high, medium, and low score? Any references?

The methodology for AMR stewardship is missing. Please add this part to the methodology.

Figure 3: Please check the spelling of macrolide. Did the Authors use the class name of antibiotics (Beta-lactams, quinolone, macrolide, etc. in the questionnaire? If yes, colistin should be polymyxin. However, the question is, why did the authors use the class name? Generally, Farmers may not be able to understand an antibiotic class. In that case, how did the Authors maintain this interview?

Table 2: What is the meaning of "n"? Be sure that every table is self-explanatory.
