# Supplementary material for: Antimicrobial stewardship hindered by inadequate biosecurity and biosafety practices, and inappropriate antibiotics usage in poultry farms of Nepal–A pilot study
Source: PLoS One. 2024 Mar 1;19(3):e0296911. doi: 10.1371/journal.pone.0296911 (PMC10906820; doi:10.1371/journal.pone.0296911)
Supplement: Attachment — Submitted filename: PONE-D-23-10881_Response to Reviewers.docx [file pone.0296911.s009.docx]

**PONE-D-23-10881 – Response to the Reviewers**

The authors did a good job explaining the issues with biosafety and biosecurity measures in Nepal and how that leads to increased AMR and risk for spread of infectious diseases. In general, the study is important as it highlights some of the issues of global health importance like antimicrobial resistance and inadequate biosafety and biosecurity measures. I have included here comments that the authors can use to improve their manuscript.

**Title:** This is not a major issue, but the title can be rephrased for clarity.

One example: Antimicrobial stewardship hindered by inadequate biosecurity and biosafety practices, and inappropriate antibiotics usage in poultry farms of Nepal.

**ANS** – Title has been rephrased.

**INTRODUCTION:**

The authors do not clearly show the identified gaps they wanted to address through this study. I think that the last paragraph could include a few more details to explicitly indicate:

- The gaps in knowledge in relation to biosafety and biosecurity.
- Specific research questions they sought to address in this study.

**ANS** – Introduction section has been reworked.

**METHODOLOGY:**

It is understood that the authors wanted to conduct "a comprehensive risk assessment and status evaluation of biosafety, biosecurity and AMR stewardship". The study design should be revised to indicate how each component of the methodology is addressing the stated research question/specific objectives. The following points should be made clear in their study design:

Choice of farms:

In the introduction the authors show that contributions of commercial and backyard are in comparable proportions (56% vs 46%) in Nepal. It would be important to investigate both types of operations. The authors should indicate if this was considered in their study design (or why not that was not the case).

**ANS** – addressed in lines 118-120 under ‘Methodology’.

How many farms are in each district and how were the four farms selected?

**ANS** – due to budgetary constraints and to ensure uniformity among the sampling sites, four small-medium farms were selected in each district. This is a limitation of the study.

Were these broiler or layer farms?

**ANS** – Two broiler and two layer farms were selected in each district. This details has been added in methodology as well. A table has been added with farm details (Table 1).

Choice of organisms to investigate:

What research question did the authors want to address by collecting samples and detecting pathogens by PCR? Although not explicitly stated, it can be understood that this is part of "status evaluation of biosafety, biosecurity and AMR stewardship". The authors chose only viral pathogens and two Mycoplasma species. Some bacteria like *Salmonella*, *E. coli*, *Avibacterium paragallinarum*, ... could have been a good indicator or AMR status since antibiotic usage and AMR are a major part of the study. There might be reasons why these were not included. These should be stated and indicated as one of the study limitations.

**ANS** – Indeed, and it is a limitation of the study but the viral and mycoplasma pathogens were selected based on Biovac Nepal’s experience with commercial diagnostics in Nepal. The pathogens selected were detected the most in commercial farms that Biovac had screened for its commercial activities. There is certainly a selection bias and is noted as a limitation in conclusion of the Discussion.

**Additional comments**

Line 114: Were they any criteria for selection of the 30 birds for sampling?

**ANS** – We utilized the practicality of the central limit theorem and selected 30 birds for sampling. It has been mentioned in methodology [lines 123-126].

Lines 116-117: The pooling process is not clear. Was the pooling based on any criteria like age, sex, ...? Showing total numbers before and after pooling will help to understand the pooling process.

**ANS** – As we were screening for infectious diseases, we expected the 30 chicken samples from each farm to be relatively homogenous in terms of exposure or infection status. Form each farm, pooling the oral and cloacal swabs would ensure representative data while reducing the number of tests. The objective of the study was to identify infections in the farms as a whole and not determine prevalence of the disease. Thus the samples were pooled together.

Line 126: The authors indicate that they designed their own primers instead of using published ones. This could be warranted if they had reasons to believe that the organisms in the study area may be different from those used in published literature. In that case, they need to show the source of the genome/gene sequences that were used to design the primers and if possible/applicable, indicate the sequence accession numbers in in the public databases.

**ANS** – A column has been added to the Table 2 with the GenBank accession numbers or the citations used for the reference sequences.

Lines 147 -148: Did the authors need to use a subtitle if there is only one section?

**ANS** – the subtitle has been removed.

These two statements seem to contradict each other. The authors should clarify that:

Lines 171-172: Only two farms implemented one out of four record keeping practices.

Lines 178-179: Similarly, record keeping was implemented in all but four farms.

**ANS** – Line 196 - ‘Only two farms implemented one out of four record keeping practices’ has been changed to ‘Only two farms implemented one out of four **rodent control** practices.’

Discussion: It would be good if the authors can mention a few strengths AND/OR limitations of the study and what gaps still need to be addressed by research.

**ANS** – this has been added in Discussion

**Grammar and typographical errors**

There are several grammar and typographical errors. Below are a few that I picked but the authors should make a thorough search of those. An electronic grammar checker can be helpful here.

**ANS** – the grammar and typographical errors have been fixed

Line 24: Threatening instead of threating.

Line 27: Questionnaires in plural.

Line 33: Scores instead of scored; **For** personnel instead of **by** personal ....

Lines 33, 175 & 182: Should it be **personnel** instead of **personal** for consistency with lines 28, 153, 156.

/

Lines 34 & 192: Simplify for clarity: why not "At least one of the screened pathogens was ..."

Line 35: pathogen instead of disease.

Line 48: Chickens in plural?; with only **a** small number.

line 49: Almost half of the poultry production (46%) comes from....

Line 58: Write HPAI in full for the first appearance of the acronym.

Line 64: Poultry production**s** ....

Line 73: Capitalize and italicize *Salmonella*.

Line 99: The sampling and survey **were** ....

Line 184: Use a simple title. Something like "Biosafety and Biosecurity risk assessment results"

Lines 186 & 187: I would suggest the authors to find a different way to highlight the cells that indicated by color-codes or consult with the editorial team for advice as colors may not be seen in tables.

**ANS** – the colors have been removed and the text is written in Bold.

Lines 212-215: These are antibiotic families not individual antibiotics. For example, on line 213, I believe that they meant "Tetracyclines were the most used (36%) antibiotics" OR "Tetracycline was the most used (36%) family of antibiotics."

**ANS** – fixed in manuscript [lines – 239-240]

Figure 1: the figure does not clearly show the geographical relationship between the city of …. and the study sites. The city boundaries would be a good addition. If necessary, a plain map could be used instead of the terrain map. Does the scale bar apply to the top or bottom map?

**ANS** – An updated map has been uploaded
