# Supplementary material for: Antimicrobial stewardship hindered by inadequate biosecurity and biosafety practices, and inappropriate antibiotics usage in poultry farms of Nepal–A pilot study
Source: PLoS One. 2024 Mar 1;19(3):e0296911. doi: 10.1371/journal.pone.0296911 (PMC10906820; doi:10.1371/journal.pone.0296911)
Supplement: Attachment — Submitted filename: Response to Reviewers_Oct 2023.docx [file pone.0296911.s011.docx]

PONE-D-23-10881R1

**Reviewers' comments**

Please use the space provided to explain your answers to the questions above. You may also include additional comments for the author, including concerns about dual publication, research ethics, or publication ethics. (Please upload your review as an attachment if it exceeds 20,000 characters)

**Reviewer #2: The authors addressed the comments made previously, but the categorization of antibiotics still needs to be revised. For example, β lactams include both penicillins and cephalosporins. Again colistin is individual polymyxin. So, line 240-243 can be revised as follows : The farmers were also inquired about various types and proportion of antibiotics they used (Figure 3). Tetracyclines were the most used (36%) antibiotic class, followed by polymyxins (colistin) (14%), quinolones (12%), aminoglycosides (12%) and macrolides (9%). Penicillins (3%) and cephalosporins (2%) were the least used antibiotic classes.
For clarification -** [**https://www.woah.org/app/uploads/2021/06/a-oie-list-antimicrobials-june2021.pdf**](https://www.woah.org/app/uploads/2021/06/a-oie-list-antimicrobials-june2021.pdf)**.**

**ANS –** Changes have been made in the manuscript. Penicillins and cephalosporins have been combined into beta-lactams. Image has also been changed.

**Line 283 : …… polymyxin (colistin)……**

**ANS –** Changes have been made in the manuscript

**Fig. 3
Consider revising classes of the antibiotics - Tetracycline, polymyxin (colistin), quinolone, aminoglycoside, macrolide, sulfonamide, pleuromutilin, penicillin, cephalosporin.**

**ANS –** Changes have been made in the manuscript

**The title of the manuscript requires a change. The Authors used biosafety in the title and throughout the manuscript. According to WHO, Biosafety is the safe working practices associated with handling of biological materials. This is related to laboratories or any area where biological materials particularly, pathogens are handled. Therefore, to me, biosafety is not associated with poultry farms. I suggest erasing the word biosafety from the title as well as the whole manuscript.**

**ANS –** According to the CDC, biosafety not only refers to handling of biological materials but also containment of potentially hazardous biological agents to reduce the risk of exposure to personnel or the environment. This could be achieved by using combination of protective barriers like using PPE, appropriate equipment, and conducting risk assessments (Meechan, P. J., & Potts, J. (2020). Biosafety in microbiological and biomedical laboratories.). Therefore, we believe usage of the term is appropriate as inadequate practices in poultry farms can lead to exposure of pathogens to either humans or the environment.

Additionally, the term biosafety is commonly been used in several publications that address similar issues mentioned in this paper. Few are mentioned below:

Hedman, H. D., Vasco, K. A., & Zhang, L. (2020). A review of antimicrobial resistance in poultry farming within low-resource settings. *Animals*, *10*(8), 1264.

Bello, O. G., Abdulrahaman, O. L., Kayode, A. O., Busari, I. Z., & Koloche, I. M. (2022). Awareness of poultry farmers on biosafety practices against infectious diseases in Kano State, Nigeria. *Journal of Agricultural Extension*, *26*(2), 1-10..

Sulzbach, A., Ferla, N. J., da Silva, G. L., & Johann, L. (2022). World occurrence and related problems caused by Megninia ginglymura (Mégnin)(Acari: Analgidae) in commercial poultry farms–a review. *World's Poultry Science Journal*, *78*(1), 215-229.

**General comment: Minor English collections are required, particularly, grammar and spelling checks. The table and figure should be self-explanatory**

**ANS –** Changes have been made to captions for Table 1 and 2 to provide better explanations.

**Species should be italicized.**

**ANS –** All species have been italicized

**Please check the references are appropriately selected. Find the other corrections and clarifications below.
Line No. 22-24: Please check the sentence, particularly this part, “can complicate the spread of poultry diseases…”. Maybe it will be, “can enhance the spread of poultry diseases….”**

**ANS –** this has been changed in the manuscript

**Line no. 70-71: Please mention, for which animal feed unprocessed poultry waste is being used and contributes to the spread of AI. Please check the reference (13) as well for this statement in line no. 70-71.**

**ANS –** The statement has been edited and appropriate citation has been added.

**Line no. 73: Please check the format of writing Escherichia coli and make the first letter of salmonella as capital.**

**ANS –** changed in the manuscript

**Line no. 86-87: The Authors have justified the cause of the sampling area around the city of Kathmandu. They used a map showing the sample collection area (four districts, including the farms in the districts) for a better understanding of how the city is surrounded by sample collection areas. I suggest adding the Figure 1 in the methods sections.**

**ANS –** Figure 1 is in the Methodology section.

**Line no. 106-107: Marek’s disease virus detection from feather follicle epithelial cells is best. There are some other important samples like chicken dust, bedding material, etc. Why the Authors screened Marek’s disease virus from cloacal and oropharyngeal swabs?
ANS –** This was done to maintain uniformity in sampling and we have successfully detected Marek’s disease in diagnostic samples from cloacal and oropharyngeal swabs numerous times prior to and after in the poultry sampled in this study.

**Please mention the age of every farm selected for sample collections.**

**ANS –** This has been added in Table 1.

**Line no. 151-152: What is the basis of the categorization as high, medium, and low score? Any references?**

**ANS –** No, we created the scoring matrix.

**The methodology for AMR stewardship is missing. Please add this part to the methodology.**

**ANS –** We had developed a questionnaire for assessing AMR stewardship practices and it is mentioned in methodology (line – 124)

**Figure 3: Please check the spelling of macrolide. Did the Authors use the class name of antibiotics (Beta-lactams, quinolone, macrolide, etc. in the questionnaire? If yes, colistin should be polymyxin. However, the question is, why did the authors use the class name? Generally, Farmers may not be able to understand an antibiotic class. In that case, how did the Authors maintain this interview?**

**ANS** – Spelling of macrolide has been corrected and colistin has been changed to polymyxin (changes have been made to the manuscript as well).

We asked the names of individual brand of antibiotics used by the farmers and grouped them according to their class as different antibiotics of the same classes were used in different farms. Thus, to avoid confusion, we used class of those antibiotics in the manuscript.

**Table 2: What is the meaning of “n”? Be sure that every table is self-explanatory.**

**ANS –** N stands for nucleoprotein – it has been added to the manuscript. Caption of the table has also been edited.
